# Supplementary material for: Disordered regions in the IRE1α ER lumenal domain mediate its stress-induced clustering
Source: EMBO J. 2024 Sep 4;43(20):12. doi: 10.1038/s44318-024-00207-0 (PMC11480506; doi:10.1038/s44318-024-00207-0)
Supplement: Supplementary file 1 — Appendix [file 44318_2024_207_MOESM1_ESM.pdf]

Appendix Table of Contents

Appendix Table S1, page 2
Appendix Table S2, page 3
Appendix Table S3, page 4
Appendix Table S4, page 5
Appendix Figure S1, page 6
Appendix Figure S2, page 7
Appendix Figure S3, page 8
Appendix Figure S4, page 9

Appendix Table S1. **mCherry-IRE1 LD-10His** Fluorescence Intensity on the SLBs.

Concentration of Ni-NTA Lipid (mol %)	Concentration of mCherry-IRE1 LD-10His	Fluorescence Intensity (AU)
1%	50 nM	2655.90
1%	200 nM	9267.75
1%	500 nM	12421.96
1%	1 μM	13245.89
2%	50 nM	4994.50
2%	200 nM	13733.22
2%	500 nM	18802.05
5%	1 μM	27346.64

Appendix Table S2. Fits of the FRAP curves obtained from SLB tethered mCherry- IRE1α LD-10His and Atto488-DPPE. The fits are linked to the data in **Fig. 1** and **EV. 1 and EV2.**

A. Fits of the FRAP curves of **mCherry-hIRE1 LD-10His** on SLBs in **Fig 1F-I**

mCH	**IRE1 LD**	**+ MPZ1N**	**+MPZ1N-2X**	**+ MPZ1N-2X-RD**
**Fit values y = a*(1-exp(-b*x))**				
Mobile fraction [%]	76.48 ± 14.45	89.77 ± 3.07	66.80 ± 4.52	81.93 ± 14.32
Half-life time [s]	37.64 ± 6.64	27.60 ± 4.51	83.25 ± 24.00	32.95 ± 6.81
Diffusion coefficient [μm^2/s]	0.08 ± 0.04	0.12 ± 0.02	0.03 ± 0.01	0.09 ± 0.04

B. Fits for the FRAP curves of **Atto488-DPPE** lipids within SLBs in **EV.1A, Appendix Figure S1,** where **mCherry-hIRE1 LD-10His** is attached in **Fig 1F-I**

	Atto488-DPPE	+MPZ1N	+MPZ1N-2X	+MPZ1N-2X-RD
**Fit values y = a*(1-exp(-b*x))**				
Mobile fraction [%]	97.25 ± 2.28	93.53 ± 3.17	96.30 ± 1.96	94.18 ± 2.87
Half-life time [s]	2.78 ± 0.5	2.36 ± 0.23	2.95 ± 0.88	2.35 ± 0.52
Diffusion coefficient [μm^2/s]	1.04 ± 0.41	1.22 ± 0.47	0.95 ± 0.14	1.21 ± 0.39

C. Fits for the FRAP curves of **mCherry-hIRE1 LD-10His** on SLBs in the absence and presence of various PEG concentrations **EV.1B**

	0%PEG	8%PEG	9%PEG	10%PEG	11%PEG	12%PEG
**Fit values y = a*(1-exp(-b*x))**						
Mobile fraction [%]	69.70	57.00	46.00	23.2	11.9	nan
Half-life time [s]	25.20	47.31	87.67	126.80	291.39	nan
Diffusion coefficient [μm^2/s]	0.18	0.10	0.05	0.04	0.02	nan

D. Fits for the FRAP curves of **Atto488-DPPE** within SLBs in the absence and presence of various PEG concentrations**. EV.1C**

	0%PEG	8%PEG	9%PEG	10%PEG	11%PEG	12%PEG
**Fit values y = a*(1-exp(-b*x))**						
Mobile fraction [%]	97.4	96.8	96.6	96.4	96.3	96.2
Half-life time [s]	2.20	2.57	2.76	2.65	3.00	2.63
Diffusion coefficient [μm^2/s]	2.09	1.78	1.66	1.73	1.53	1.74

E. Fits for the FRAP curves of **Atto488-DPPE** lipids and **mCherry-10His** control on SLBs. **EV. 1E,F**

	Membrane **Atto488-DPPE**		Control Protein **mCherry-10XHis**	
	-	**+ 11%PEG**	-	**+ 11%PEG**
**Fit values y = a*(1-exp(-b*x))**				
Mobile fraction [%]	93.07 ± 3.84	98.07 ± 1.11	94.57 ± 2.91	95.53 ± 1.40
Half-life time [s]	1.26 ± 0.07	1.77 ± 0.32	10.38 ± 2.88	15.47 ± 4.93
Diffusion coefficient [μm^2/s]	3.63 ± 0.20	2.65 ± 0.50	0.47 ± 0.14	0.32 ± 0.09

Appendix Table S3. Fits of the FRAP curves of **mCherry-IRE1 LD-10His** in condensates formed in solution. The fits are linked to the data shown in **Fig. 2C,D, EV. 2L,M.**

	hIRE1 LD	hIRE1 LD + MPZ1N (1:1)	hIRE1 LD + MPZ1N (2:1)	hIRE1 LD + MPZ1N-2X (2:1)	hIRE1 LD + MPZ1N-2X (4:1)	hIRE1 LD + MPZ1N-2X-RD (2:1)	hIRE1 LD + MPZ1N-2X-RD (4:1)
**Best-fit values**							
Plateau	0.8190	0.8138	0.9307	0.3492	0.6607	0.8577	0.7212
Tau	243.7	236.1	260.0	512.6	375.0	274.3	190.9
Half time [sec]	169.0	163.7	180.2	355.3	260.0	190.2	132.3
**95% CI (profile likelihood)**							
Plateau	0.7924 to 0.8502	0.7968 to 0.8325	0.9114 to 0.9519	0.3362 to 0.3640	0.6206 to 0.7130	0.8414 to 0.8755	0.7019 to 0.7433
Tau	221.3 to 270.6	222.2 to 251.7	245.6 to 276.0	479.5 to 550.4	327.5 to 437.6	261.1 to 288.8	173.5 to 211.4
Half-time	153.4 to 187.5	154.0 to 174.4	170.2 to 191.3	332.4 to 381.5	227.0 to 303.3	181.0 to 200.2	120.2 to 146.5
**Goodness of Fit**							
R squared	0.8296	0.9350	0.9411	0.9737	0.8298	0.9600	0.7907

Appendix Table S4. Fits of the FRAP curves of **mCherry-IRE1 LD-10His** or **mCherry-IRE1 cLD-10His** in condensates formed in solution shown in **Fig. 3G.**

	LD	cLD
**Best-fit values**		
Plateau	0.9251	0.2753
Tau	281.1	406.1
Half-time	194.9	281.5
**95% CI (profile likelihood)**		
Plateau	0.8842 to 0.9756	0.2635 to 0.2893
Tau	249.7 to 320.6	371.8 to 447.0
Half-time	173.1 to 222.2	257.7 to 309.9
**Goodness of Fit**		
R squared	0.8022	0.9316

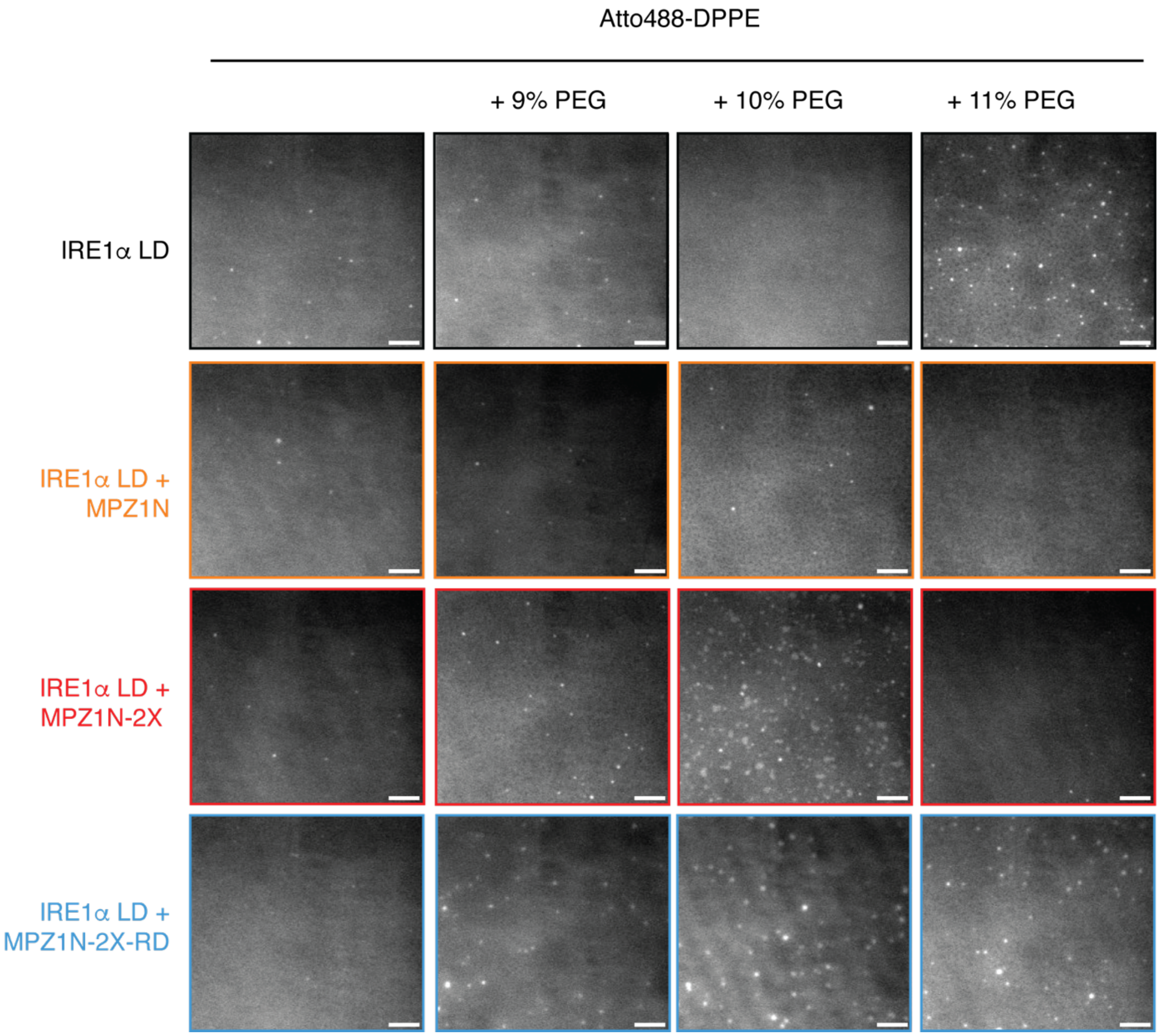

Appendix Figure S1.

A. TIRF images displaying Atto488 labeled DPPE within SLBs of experiments shown in **Fig. 1F-I**. Scale bar = 5 µm

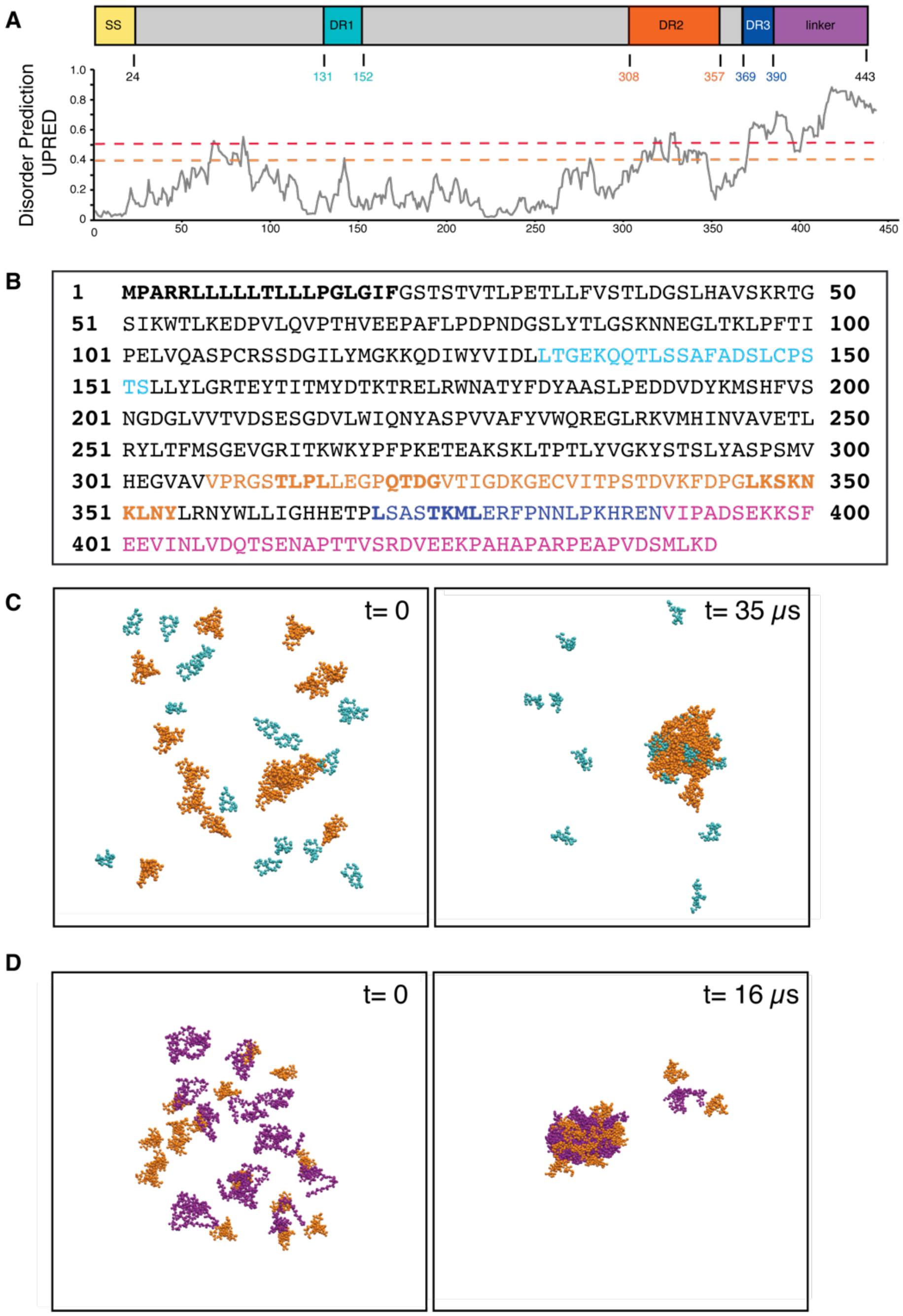

Appendix Figure S2.

A. Schematic presentation of DRs in IRE1α LD domain organization in combination with the prediction of intrinsically unstructured regions of hIRE1α LD using the IUPRED server. The red and orange lines indicate moderate and disordered propensity, respectively. **B.** Amino acid sequence of IRE1α LD where DR1, DR2 and linker segments are colored in cyan, orange and purple respectively. The signal sequence and mutated segments are highlighted in bold letters. **C**. Simulation of 16 copies of DR1 (cyan) and 16 copies of DR2 (orange). Molecular Dynamics Simulations show that the cluster formed by DR2 do not recruit DR1 segments**.** **D.** Simulation of 16 copies of DR2 (orange) and 16 copies of linker region (purple) (right two panels). Molecular Dynamics Simulations show that the cluster formed by DR2 recruit the linker segments.

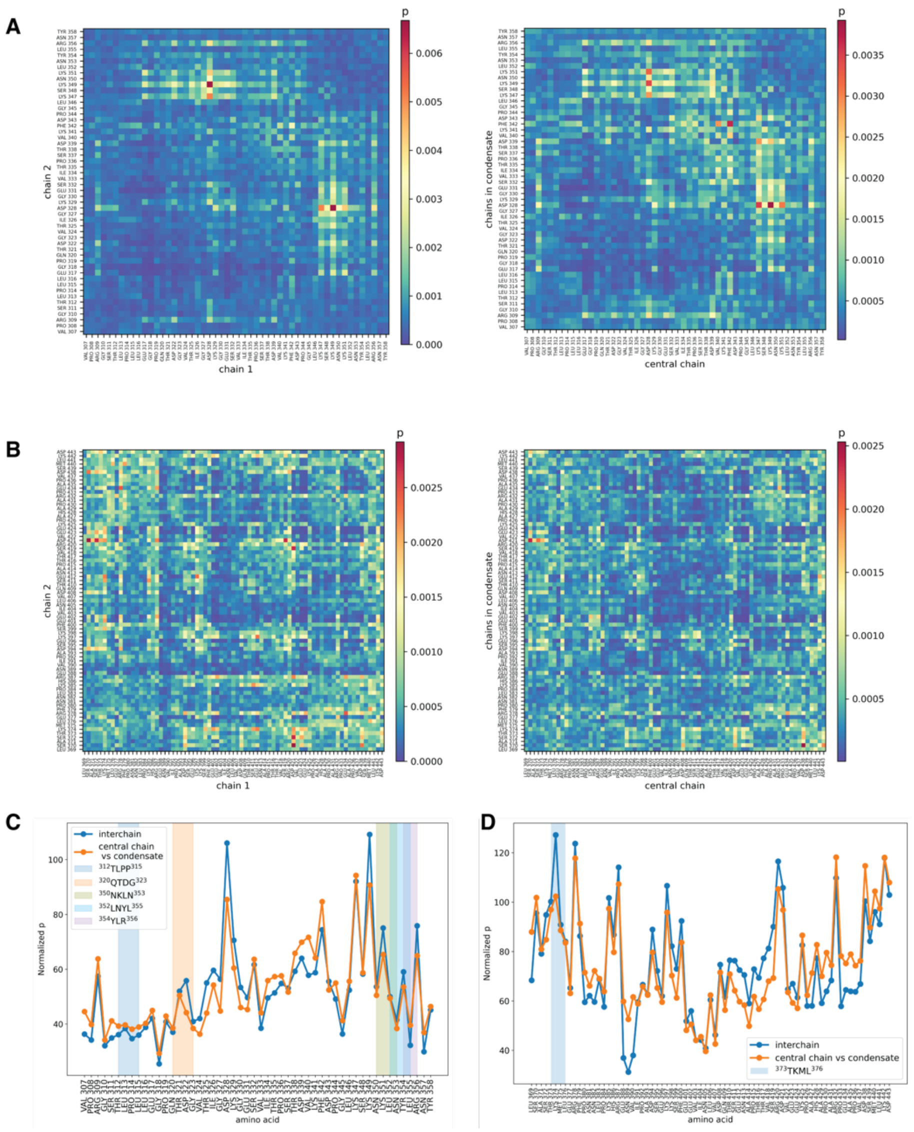

Appendix Figure S3.

A. Contact maps for interchain and condensate interactions in simulations containing two or 33 copies of DR2. **B.** Contact maps for interchain and condensate interactions in simulations containing two or 33 copies of linker**. C.** 1D-projections of the contact maps computed for the simulations containing two or 33 copies of DR2. **D.**1D-projections of the contact maps computed for the simulations containing two or 33 copies of linker.

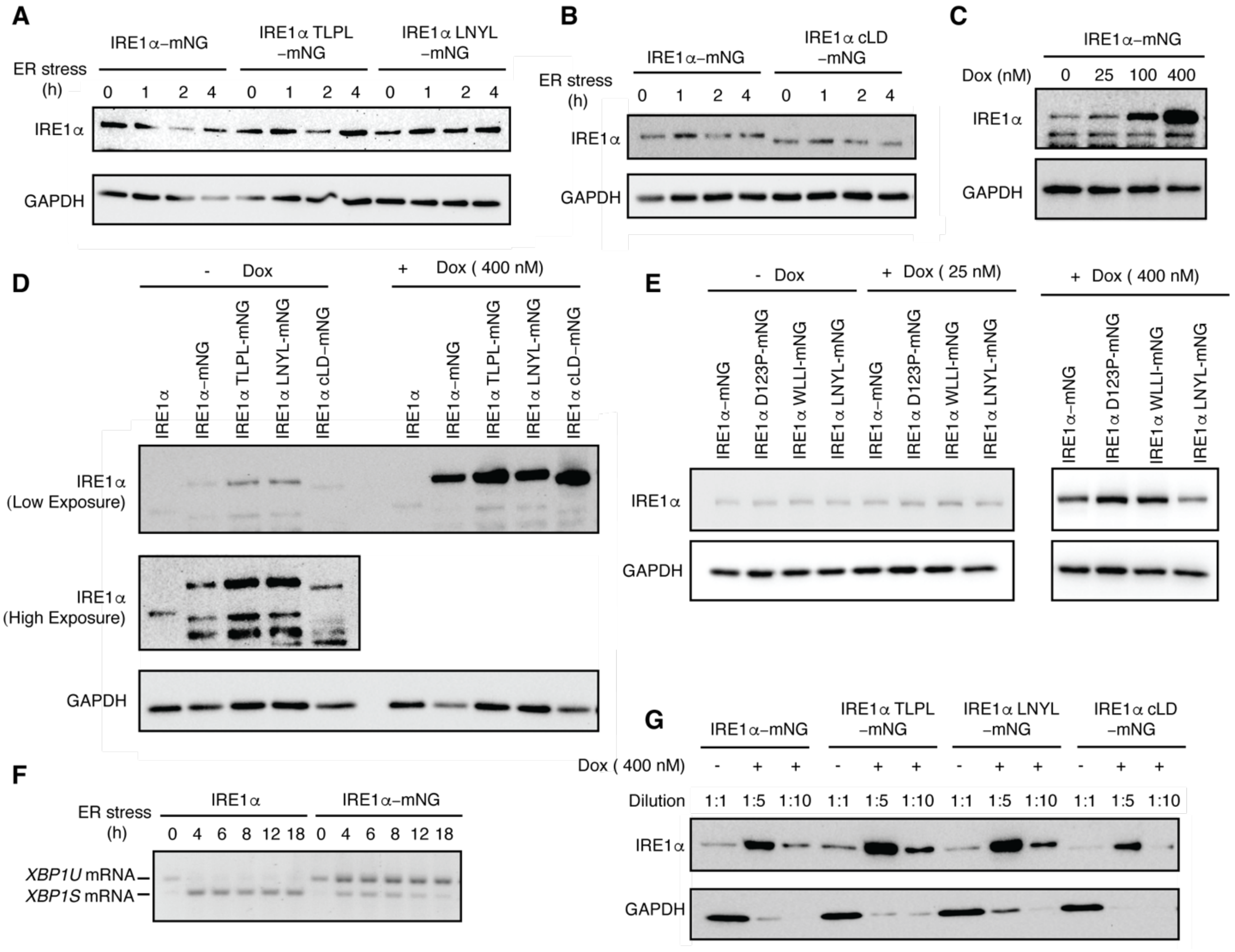

Appendix Figure S4.

A. Western blot analyses comparing the expression level of IRE1α-mNG and its mutants IRE1α TLPL-mNG and IRE1α LNYL-mNG in MEFs in the absence of doxycycline treatment at different points after induction of ER stress. **B.** Western blot analyses comparing the expression levels of IRE1α-mNG and IRE1α cLD-mNG in MEFs in the absence of doxycycline treatment at different points after induction of ER stress. **C.** Western blot analyses of the expression level of IRE1α-mNG in the absence and presence of various concentrations of doxycycline inducing its expression for 24 hours. **D.** Comparison of WT IRE1α expression levels to MEFs expressing IRE1α-mNG and its mutants in the absence and presence of 400 nM doxycycline for 24 hours.

E. Comparison of WT IRE1α expression levels to MEFs expressing IRE1α-mNG and its mutants IRE1αD123P-mNG and IRE1α WLLI-mNG in the absence and presence of either 25 nM and 400 nM doxycycline for 24 hours. **F.** Semiquantitative PCR reaction to monitor splicing of *XBP1* mRNA by IRE1α-mNG (in the absence of doxycycline) and wild type IRE1α at different time points after induction of ER stress by addition of 5 µg/ml Tunicamycin. The bands are indicated as unspliced and spliced *XBP1* variants. **G.** Western blot analyses comparing the expression levels of IRE1α-mNG and its mutants in MEFs in the absence and presence of 400 nM doxycycline treatment. Lysates obtained from 400 nM doxycycline MEFs were diluted 1 to 5 and 1 to 10.
