# Supplementary material for: Disordered regions in the IRE1α ER lumenal domain mediate its stress-induced clustering
Source: EMBO J. 2024 Sep 4;43(20):12. doi: 10.1038/s44318-024-00207-0 (PMC11480506; doi:10.1038/s44318-024-00207-0)
Supplement: Supplementary file 4 — Movie EV1 [file 44318_2024_207_MOESM4_ESM.zip › MovieEV1/Movie EV1 Legend.docx]

**Movie EV1.** mCherry-IRE1α LD-10His cluster formation on SLBs after the addition of 11% PEG. Each frame is recorded every 2 sec for a total of 32 frames
