# Supplementary material for: Disordered regions in the IRE1α ER lumenal domain mediate its stress-induced clustering
Source: EMBO J. 2024 Sep 4;43(20):12. doi: 10.1038/s44318-024-00207-0 (PMC11480506; doi:10.1038/s44318-024-00207-0)
Supplement: Supplementary file 5 — Movie EV2 [file 44318_2024_207_MOESM5_ESM.zip › MovieEV2/Movie EV2 Legend.docx]

**Movie EV2.** Fusion of mCherry-IRE1α LD-10His clusters on SLBs. The movie is recorded 10 min after induction of cluster formation by addition of 11 % PEG. Each frame is recorded every 2 sec for a total of 60 frames.
