# Supplementary material for: Disordered regions in the IRE1α ER lumenal domain mediate its stress-induced clustering
Source: EMBO J. 2024 Sep 4;43(20):12. doi: 10.1038/s44318-024-00207-0 (PMC11480506; doi:10.1038/s44318-024-00207-0)
Supplement: Supplementary file 6 — Movie EV3 [file 44318_2024_207_MOESM6_ESM.zip › MovieEV3/MovieEV3 Legend.docx]

**Movie EV3.** LLPS of IRE1α LD in solution. The movie is recorded 30 min after induction of LLPS with 6% PEG.
