# Supplementary material for: Disordered regions in the IRE1α ER lumenal domain mediate its stress-induced clustering
Source: EMBO J. 2024 Sep 4;43(20):12. doi: 10.1038/s44318-024-00207-0 (PMC11480506; doi:10.1038/s44318-024-00207-0)
Supplement: Supplementary file 7 — Expanded View Figures [file 44318_2024_207_MOESM7_ESM.pdf]

Expanded View Figures

Figure EV1. Unfolded polypeptides induce clustering of human IRE1α LD on SLBs.

(**A**) TIRF images of FRAP experiments of Atto488 labeled DPPE lipids (top) and mCherry-IRE1α LD-10His (bottom) on SLBs showing the dynamic behavior within the indicated time. Scale bar = 5 µm. (**B**) FRAP curves of mCherry-IRE1α LD-10His tethered to SLBs by 1% Ni-NTA labeled lipids. The SLBs are incubated 10 min with the indicated concentration of the crowding agent PEG before the images are taken. The mobile fraction and diffusion values are decreasing with increasing PEG concentration. (**C**) FRAP curves displaying the fluorescent intensity of Atto488 labeled DPPE lipids within SLBs treated with the indicated concentration of the crowding agent PEG over time. (**D**) TIRF images displaying mCherry-10His control and the membrane (Atto488 DPPE) with and without PEG. Scale bar = 5 µm. (**E**) FRAP curves displaying the fluorescent intensity of Atto488 labeled DPPE lipids within SLBs treated with the indicated concentration of the crowding agent PEG over time. $n = 3$ independent experiments were performed to obtain the data for the FRAP curves. The error bars represent the standard deviation. (**F**) FRAP curves displaying the fluorescent intensity of mCherry-10His control on SLBs treated with the indicated concentration of the crowding agent PEG over time. $n = 4$ independent experiments were performed to obtain the data for the FRAP curves. The error bars represent the standard deviation. (**G**) TIRF images of mCherry-hIRE1α LD-10His tethered to SLBs by 1% Ni-NTA labeled lipids in the absence of PEG, in presence of 11% PEG and where PEG is washed out from the well. Scale bar = 5 µm. (**H**) Amino acid sequences of model unfolded polypeptides MPZ1N and MPZ1-N-2X and the control non-binding derivate MPZ1N-N-2X-RD. (**I**) Fluorescence anisotropy experiments monitor the interaction of N-terminal fluorescein labeled MPZ1N-2X and its derivative MPZ1N-2X-RD with IRE1α LD. MPZ1N-2X interacts with IRE1α LD at 2 µM affinity, whereas the MPZ1N-2X-RD is impaired in binding. $n = 2$ independent anisotropy experiments were performed to obtain the data for the curves. The error bars represent the standard deviation. (**J**) Diagram summarizing mCherry-IRE1α LD-10His clustering on SLBs in the presence of peptides at various PEG concentrations. "X" depicts no cluster and "O" cluster formation. (**K**) FRAP curves of mCherry-IRE1α LD-10His on SLBs in the absence (black curve) and presence of 10 µM MPZ1N (orange curve), 1 µM MPZ1N-2X (red curve) and 1 µM MPZ1N-2X-RD (blue curve) peptides. Curve marks show the mean value, error bars display the standard deviation and the values are fitted to a one-phase association curve. $N = 3$ independent experiments were performed. The error bars represent the standard deviation. (**L**) FRAP curves illustrate the mobility of Atto488 labeled DPPE lipids within the SLB belonging to the conditions in (**K**). The color code corresponds to the one used in (**K**). $n = 3$ independent experiments were performed to obtain the data for the FRAP curves. The error bars represent the standard deviation.

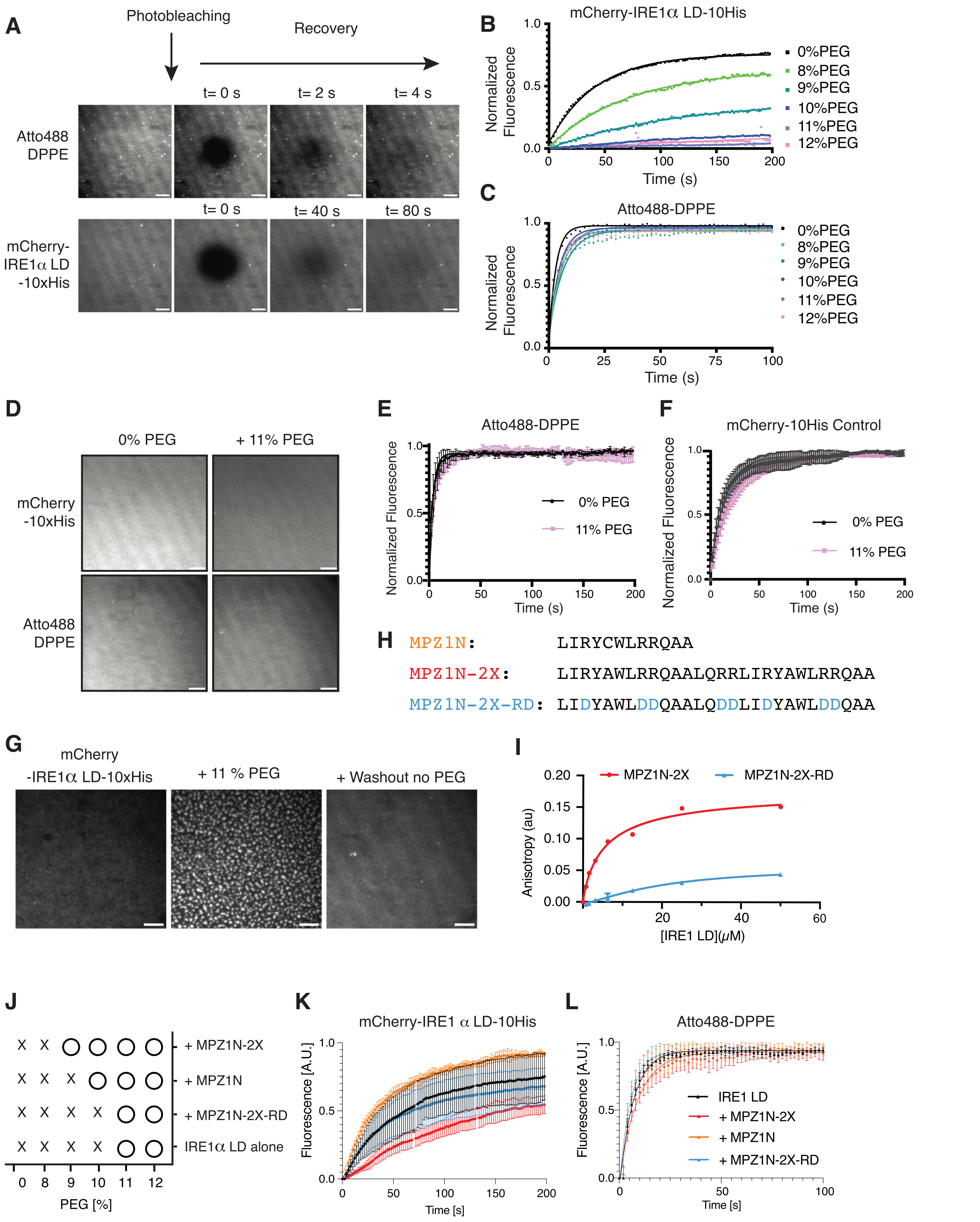
A
Photobleaching
Recovery
t= 0 s
t= 2 s
t= 4 s
Atto488 DPPE
t= 0 s
t= 40 s
t= 80 s
mCherry-IRE1α LD -10xHis
B
mCherry-IRE1α LD-10His
Normalized Fluorescence
Time (s)
0%PEG
8%PEG
9%PEG
10%PEG
11%PEG
12%PEG
C
Atto488-DPPE
Normalized Fluorescence
Time (s)
0%PEG
8%PEG
9%PEG
10%PEG
11%PEG
12%PEG
D
0% PEG
+ 11% PEG
mCherry -10xHis
Atto488 DPPE
E
Atto488-DPPE
Normalized Fluorescence
Time (s)
0% PEG
11% PEG
F
mCherry-10His Control
Normalized Fluorescence
Time (s)
0% PEG
11% PEG
H
MPZ1N: LIRYCWLRRQAA
MPZ1N-2X: LIRYAWLRRQAALQRRLIRYAWLRRQAA
MPZ1N-2X-RD: LIDYAWLDDQAALQDDLIDYAWLDDQAA
G
mCherry -IRE1α LD-10xHis
+ 11 % PEG
+ Washout no PEG
I
MPZ1N-2X
MPZ1N-2X-RD
Anisotropy (au)
[IRE1 LD](μM)
J
+ MPZ1N-2X
+ MPZ1N
+ MPZ1N-2X-RD
IRE1α LD alone
PEG [%]
K
mCherry-IRE1 α LD-10His
Fluorescence [A.U.]
Time [s]
L
Atto488-DPPE
Fluorescence [A.U.]
Time [s]
IRE1 LD
+ MPZ1N-2X
+ MPZ1N
+ MPZ1N-2X-RD

 © The Author(s)

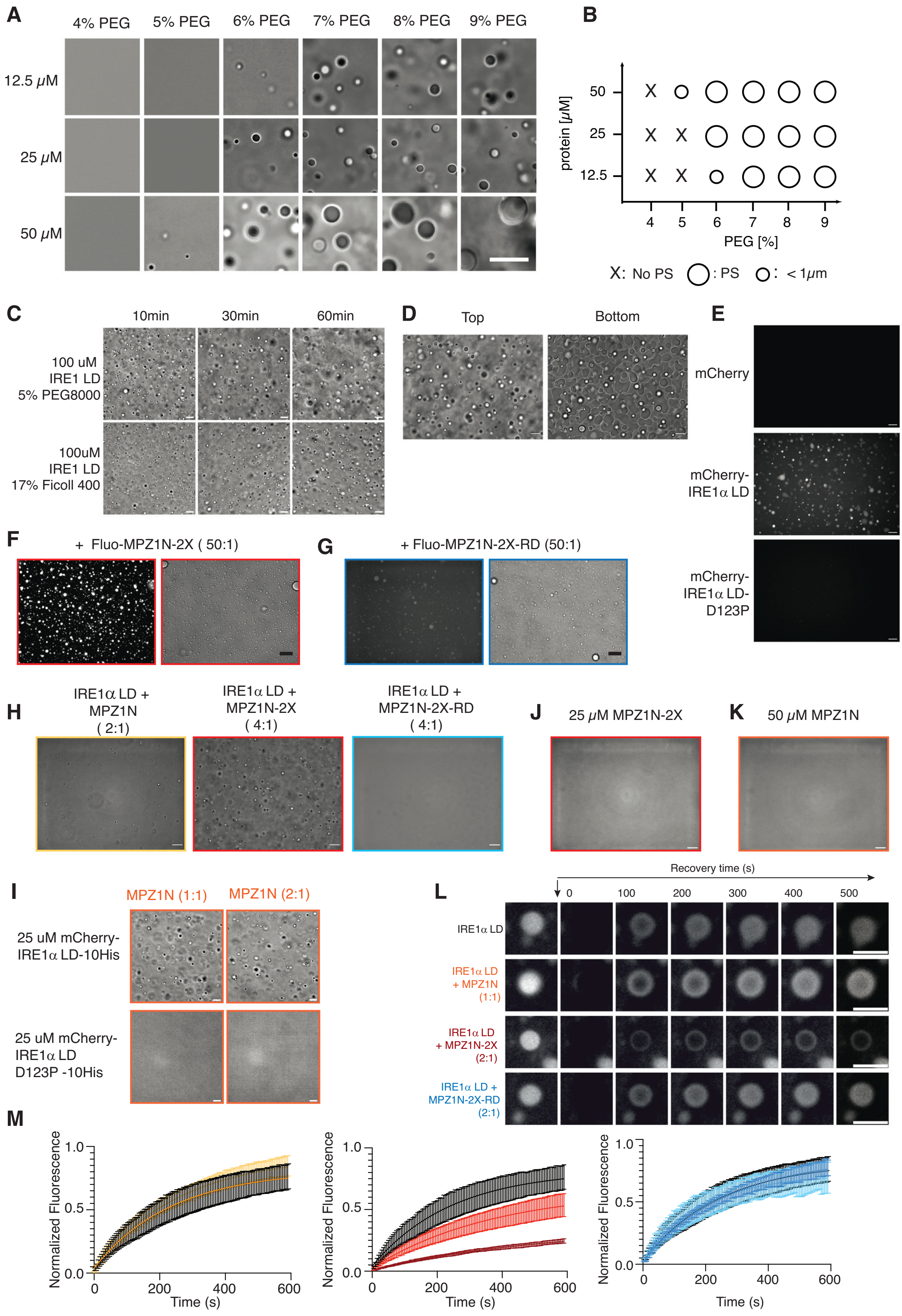
A
4% PEG
5% PEG
6% PEG
7% PEG
8% PEG
9% PEG
12.5 μM
25 μM
50 μM
B
protein [μM]
50
25
12.5
4 5 6 7 8 9
PEG [%]
X: No PS
: PS
: < 1μm
C
10min
30min
60min
100 uM IRE1 LD 5% PEG8000
100uM IRE1 LD 17% Ficoll 400
D
Top
Bottom
E
mCherry
mCherry-IRE1α LD
mCherry-IRE1α LD-D123P
F
+ Fluo-MPZ1N-2X (50:1)
G
+ Fluo-MPZ1N-2X-RD (50:1)
H
IRE1α LD + MPZ1N (2:1)
IRE1α LD + MPZ1N-2X (4:1)
IRE1α LD + MPZ1N-2X-RD (4:1)
J
25 μM MPZ1N-2X
K
50 μM MPZ1N
I
MPZ1N (1:1)
MPZ1N (2:1)
25 uM mCherry-IRE1α LD-10His
25 uM mCherry-IRE1α LD D123P -10His
L
Recovery time (s)
0 100 200 300 400 500
IRE1α LD
IRE1α LD + MPZ1N (1:1)
IRE1α LD + MPZ1N-2X (2:1)
IRE1α LD + MPZ1N-2X-RD (2:1)
M
Normalized Fluorescence
1.0
0.5
0.0
0 200 400 600
Time (s)

Figure EV2. Unfolded polypeptide-binding stabilizes human IRE1α LD condensates.

(**A**) DIC images of IRE1α LD representing the phase diagram of IRE1α LD. Scale bar = 10 μm. (**B**) Schematic phase diagram of IRE1α LD condensates at 12.5, 25 and 50 μM at 30 min incubation with 4–9% PEG as in (**A**). No phase separation (PS) is indicated by a cross and phase separation (PS) is indicated by a circle. The smaller circle refers to condensates with diameter <1 μm. (**C**) DIC images of 100 μM IRE1α LD in the presence of 5% PEG (top) or 17% Ficoll 400. Scale bar = 10 μm. (**D**) DIC images of 50 μM IRE1α LD incubated with 6% PEG for 30 min. The images are obtained at the bottom or top of the well. Scale bar = 10 μm. (**E**) Fluorescence images of 25 μM mCherry-10His control, mCherry-IRE1α LD-10His and the dimerization mutant of IRE1α LD, mCherry-IRE1α LD^{D123P}-10His after 30 min incubation with 6% PEG. Scale bar = 10 μm. (**F**) Confocal (left) and bright field (right) images displaying the recruitment of Fluorescein-labeled MPZ1N-2X (red) peptide into preformed IRE1α LD condensates. Scale bar = 13 μm. (**G**) Confocal (left) and bright field (right) images displaying the recruitment of Fluorescein-labeled MPZ1N-2X-RD (blue) peptides into preformed IRE1α LD condensates. Scale bar = 13 μm. (**H**) DIC microscopy images of 50 μM IRE1α LD incubated with MPZ1N (2:1 stoichiometry, left), MPZ1N-2X (4:1 stoichiometry, middle) or MPZ1N-2X-RD (4:1 stoichiometry, right panel) at 30 min after induction of phase separation with 5% PEG. Scale bar = 10 μm. (**I**) DIC microscopy images of 25 μM mCherry-IRE1α LD (top) or mCherry-IRE1α LD^{D123P} in the presence of 5% PEG and MPZ1N peptide at 1:1 and 1:2 molar ratio. Scale bar = 10 μm. (**J**) DIC images of 25 μM MPZ1N-2X peptide in the presence of 6% PEG. Scale bar = 10 μm. (**K**) DIC images of 50 μM MPZ1N peptide in the presence of 6% PEG. Scale bar = 10 μm. (**L**) FRAP images of a single IRE1α LD condensate in absence and presence of the model unfolded peptides at the indicated stoichiometry taken before and at the indicated time points after photobleaching. Scale bar = 5 μm. (**M**) FRAP curves of 25 μM IRE1α LD and 6% PEG in the absence (black curve) and in the presence of MPZ1N peptide (2:1 stoichiometry, light orange curve, 1:1 stoichiometry orange curve), MPZ1N-2X peptide (4:1 stoichiometry, red, 2:1 stoichiometry dark red) and MPZ1N-2X-RD control peptide (4:1 stoichiometry, light blue, 2:1 stoichiometry blue). $n = 9$ condensates in 3 independent experiments were performed to obtain the data for the FRAP curves.

 © The Author(s)

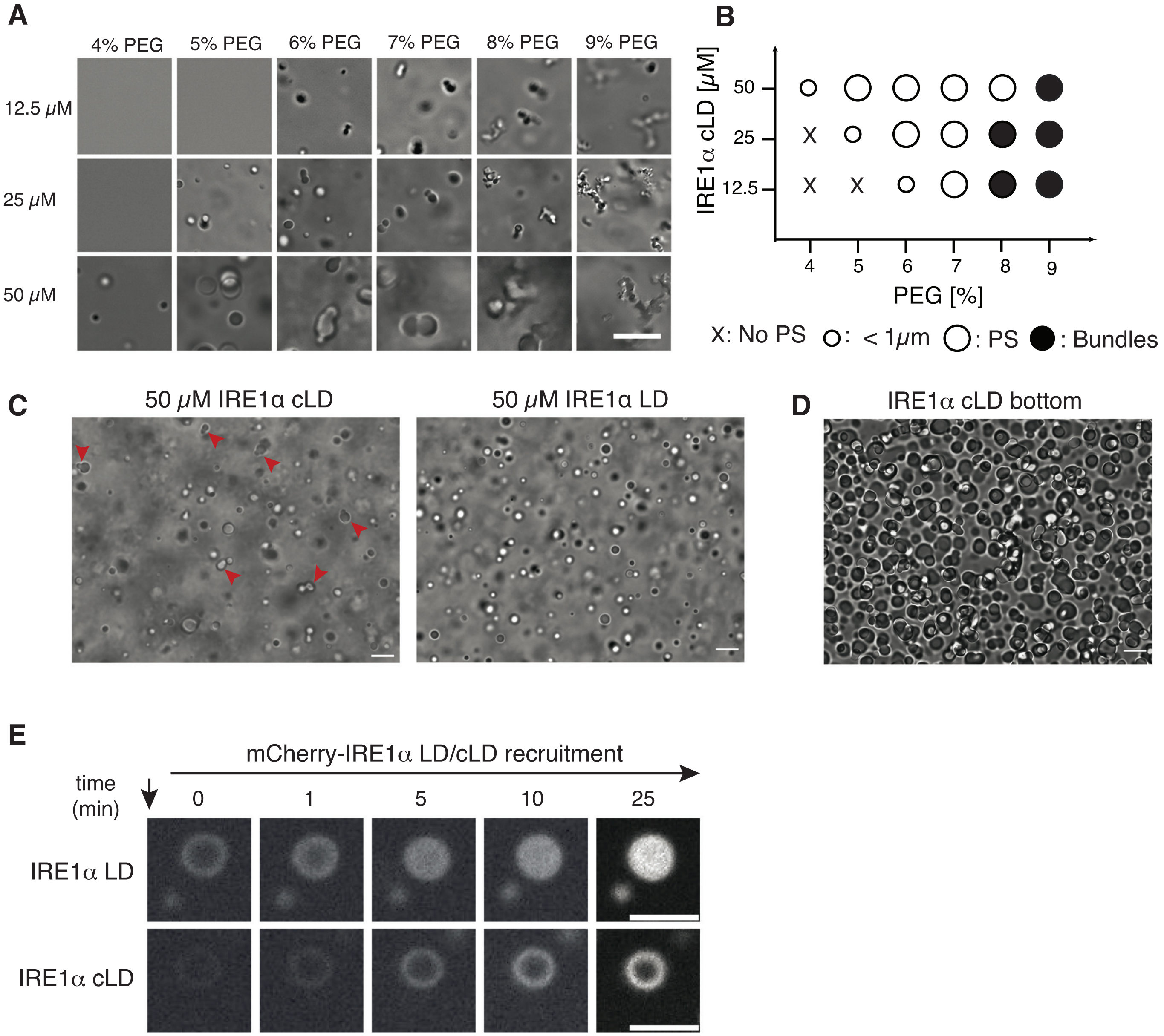

Figure EV3. Human IRE1α cLD forms rigid condensates.

(**A**) DIC Images of IRE1α cLD representing the phase diagram at 12.5, 25 and 50 μM acquired after 30 min incubation with PEG at concentrations ranging from 4 – 9%. Scale bar = 10 μm. (**B**) Phase diagram of IRE1α cLD based on images in (**A**). No phase separation (PS) is indicated by a cross, phase separation (PS) is indicated by a circle and condensates that resemble beads on a string are represented by a black circle (bundles). The smaller circle refers to smaller condensates (diameter < 1 μm). (**C**) DIC images of IRE1α cLD (left) and IRE1α LD (right), the condensates that fail to fuse are shown with red arrows. Scale bar = 10 μm. (**D**) DIC images of the bottom of the well of IRE1α cLD (50 μM) condensates taken 60 min after induction of phase separation via addition of 6% PEG showing the phase separation propensity and wetting effect. Scale bar = 10 μm. (**E**) Fluorescence images of 25 μM IRE1α LD (top) or IRE1α cLD (bottom) condensates at the indicated time points after 30 min incubation with 6% PEG following the recruitment of 2% mCherry labeled IRE1α LD or cLD, respectively. mCherry-IRE1α LD-10His is recruited to the center of preformed IRE1α LD condensates, whereas mCherry-IRE1α cLD-10His could only associate with the outer shell of the preformed IRE1α cLD condensates. Scale bar = 5 μm.

© The Author(s)

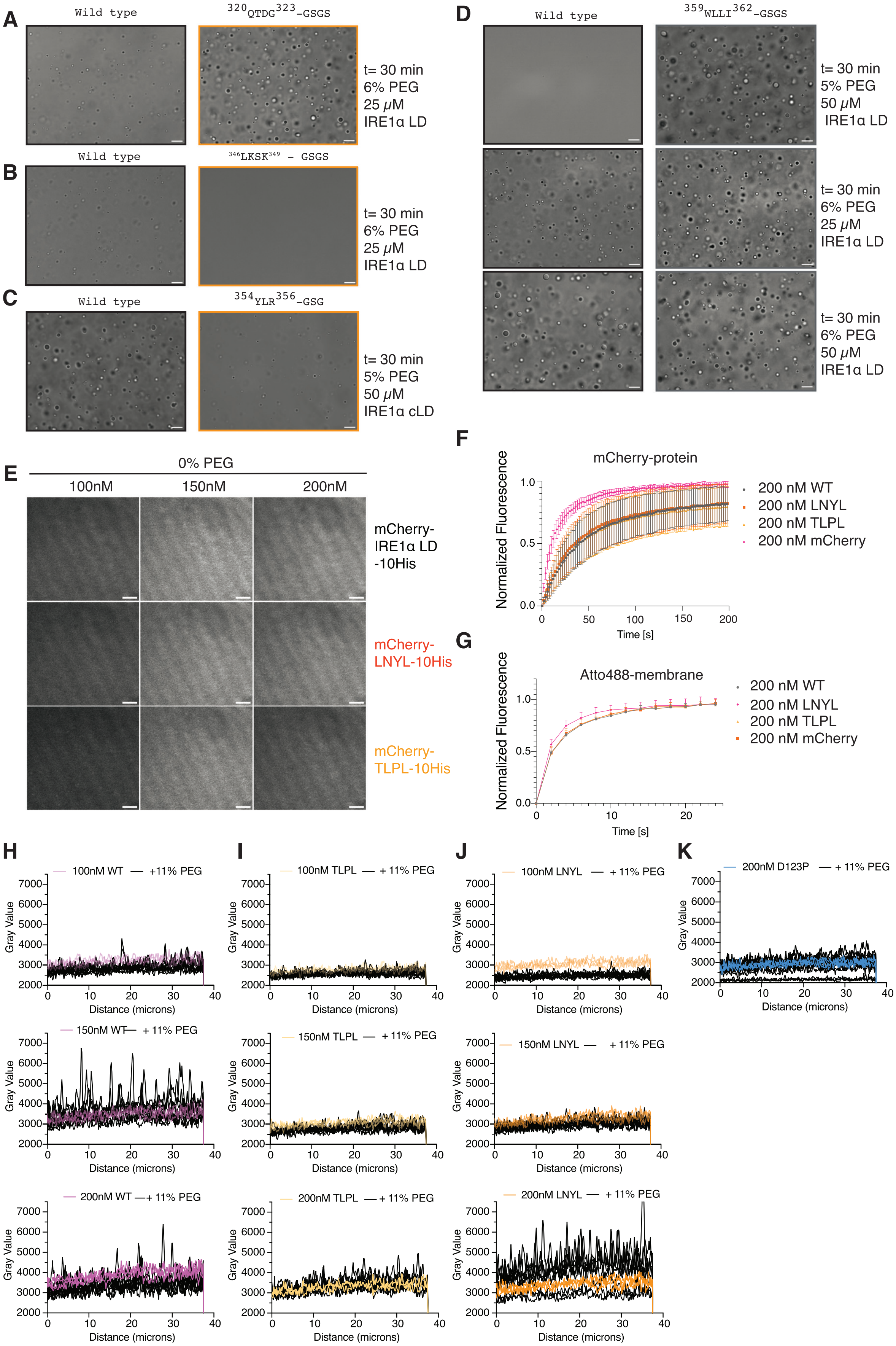
A
Wild type
320QTDG323-GSGS
t= 30 min
6% PEG
25 μM
IRE1α LD
B
Wild type
346LKSK349 – GSGS
t= 30 min
6% PEG
25 μM
IRE1α LD
C
Wild type
354YLR356-GSG
t= 30 min
5% PEG
50 μM
IRE1α cLD
D
Wild type
359WLLI362-GSGS
t= 30 min
5% PEG
50 μM
IRE1α LD
t= 30 min
6% PEG
25 μM
IRE1α LD
t= 30 min
6% PEG
50 μM
IRE1α LD
E
0% PEG
100nM
150nM
200nM
mCherry-IRE1α LD-10His
mCherry-LNYL-10His
mCherry-TLPL-10His
F
mCherry-protein
Normalized Fluorescence
Time [s]
200 nM WT
200 nM LNYL
200 nM TLPL
200 nM mCherry
G
Atto488-membrane
Normalized Fluorescence
Time [s]
200 nM WT
200 nM LNYL
200 nM TLPL
200 nM mCherry
H
100nM WT — +11% PEG
150nM WT — + 11% PEG
200nM WT — + 11% PEG
Gray Value
Distance (microns)
I
100nM TLPL — + 11% PEG
150nM TLPL — + 11% PEG
200nM TLPL — + 11% PEG
J
100nM LNYL — + 11% PEG
150nM LNYL — + 11% PEG
200nM LNYL — + 11% PEG
K
200nM D123P — + 11% PEG

© The Author(s)

Figure EV4. Mutagenesis analyses reveal the critical role of the DRs in IRE1α LD clustering.

(**A**) DIC images of 25 μM WT IRE1α LD and IRE1α LD ^{320}QTDG^{323}-GSGS mutant showing LLPS behavior 30 min after induction of phase separation by the addition of 6% PEG. (**B**) DIC images of 25 μM WT IRE1α cLD and IRE1α cLD ^{346}LKSK^{349}-GSGS mutant showing LLPS behavior 30 min after induction of phase separation by the addition of 6% PEG. (**C**) DIC images of 50 μM WT IRE1α cLD and IRE1α cLD ^{354}YLR^{356}-GSG mutant showing LLPS behavior 30 min after induction of phase separation by the addition of 5% PEG. (**D**) DIC images comparing the LLPS behavior of WT IRE1α LD (left column) and IRE1α LD ^{359}WLLI^{323}-GSGS mutant (right column) 30 min after induction of phase separation at 50 μM protein concentration and 5% PEG (top row) at 25 μM protein concentration and 6% PEG (middle row) and at 50 μM protein concentration and 6% PEG (bottom row). Scale bar for all images = 10 μm. (**E**) TIRF images of mCherry-IRE1α LD-10His (top) and the mCherry tagged mutants ^{352}LNYL^{355-}GSGS (middle) and ^{312}TLPL^{315-}GSGS (bottom) tethered to SLBs by 1% Ni-NTA labeled lipids at concentrations between 100 – 200 nM displaying an evenly distributed fluorescent signal at all concentrations. Scale bar = 5 μm. (**F**) FRAP curves of mCherry-IRE1α LD-10His, the mCherry tagged mutants ^{352}LNYL^{355-}GSGS and ^{312}TLPL^{315-}GSGS and mCherry control tethered to SLBs by 1% Ni-NTA labeled lipids at a concentration of 200 nM. Curve marks show the mean value, error bars display the standard deviation. $n = 4$ independent experiments were performed. (**G**) FRAP curves of Atto488 labeled DPPE lipids within SLBs belonging to the tethered proteins in (**E**). (**H**) Intensity plot analyses of the mCherry signal in the TIRF images in Fig. 5C for mCherry-IRE1α LD-10His at 100 (top), 150 (middle) and 200 nM (bottom) in the absence (3 lines, colored) and in the presence of 11% PEG (9 lines from three different field of views on the same membrane, black). (**I**) Intensity plot analyses of the mCherry signal in the TIRF images in Fig. 5C for mCherry-^{312}TLPL^{315-}GSGS-10His similarly displayed as in (**H**). (**J**) Intensity plot analyses of the mCherry signal in the TIRF images in Fig. 5C for mCherry-^{3352}LNYL^{355-}GSGS-10His similarly displayed as in (**H**). (**K**) Intensity plot analyses of the mCherry signal in the TIRF images in Fig. 5D for mCherry-D123P-10His at 200 nM in the absence (3 lines, blue) and in the presence of 11% PEG (9 lines from three different field of views on the same membrane, black).

© The Author(s)

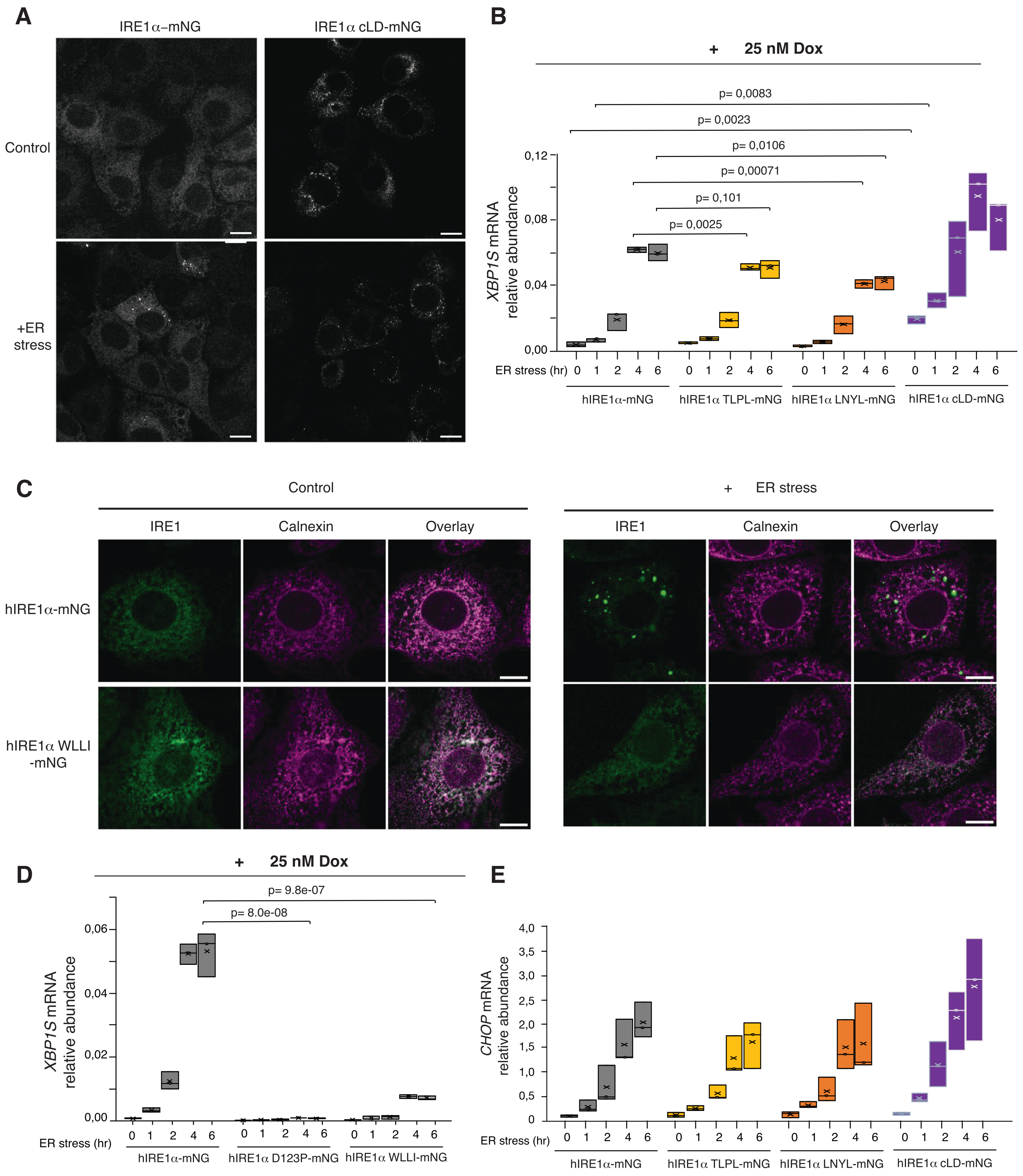
A
IRE1α–mNG
IRE1α cLD-mNG
Control
+ER stress
B
+ 25 nM Dox
p= 0,0083
p= 0,0023
p= 0,0106
p= 0,00071
p= 0,101
p= 0,0025
XBP1S mRNA relative abundance
0,12
0,08
0,04
0,00
ER stress (hr) 0 1 2 4 6
hIRE1α-mNG
hIRE1α TLPL-mNG
hIRE1α LNYL-mNG
hIRE1α cLD-mNG
C
Control
+ ER stress
IRE1
Calnexin
Overlay
hIRE1α-mNG
hIRE1α WLLI -mNG
D
+ 25 nM Dox
p= 9.8e-07
p= 8.0e-08
XBP1S mRNA relative abundance
0,06
0,04
0,02
0,00
ER stress (hr) 0 1 2 4 6
hIRE1α-mNG
hIRE1α D123P-mNG
hIRE1α WLLI-mNG
E
CHOP mRNA relative abundance
4,0
3,5
3,0
2,5
2,0
1,5
1,0
0,5
0
ER stress (hr) 0 1 2 4 6
hIRE1α-mNG
hIRE1α TLPL-mNG
hIRE1α LNYL-mNG
hIRE1α cLD-mNG

 © The Author(s)

Figure EV5. IRE1α LD DR mutants display impaired IRE1α clustering and activity in vivo.

(**A**) Immunofluorescence images of MEFs treated with 100 nM doxycycline to induce expression of IRE1α-mNG and the IRE1α cLD-mNG mutant in the absence (top row) of stress and treated with 5 μg/ml ER stressor Tunicamycin for 4 h (bottom row). IRE1α-mNG and its mutants are visualized by mNG fluorescence (green). Scale bar = 10 μm. (**B**) qRT-PCR to monitor splicing of *XBP1* mRNA by IRE1α-mNG and its mutants at different time points after induction of ER stress by addition of 5 μg/ml Tunicamycin. Cells are treated with 25 nM doxycycline for 24 h to induce expression of IRE1α-mNG and its mutants. Displayed are the values of $n = 3$ independent experiments and the *P* value. The *P* values were determined by a two-sided Student's *t*-test. The data are shown as box plots. The central line in the box plot marks the median and the cross shows the mean, the boxes mark the first and third quartiles and the whiskers display the maximum and the minimum points. (**C**) Immunofluorescence images of MEFs treated with 400 nM doxycycline expressing IRE1α-mNG or its mutants in the absence (left panel) of stress and treated with 5 μg/ml Tunicamycin for 4 h (right panel). IRE1α-mNG and its mutants are visualized by mNG fluorescence (green) and the ER-chaperone Calnexin is stained by anti-calnexin antibody (purple). Scale bar = 10 μm. (**D**) qRT-PCR to monitor splicing of *XBP1* mRNA by IRE1α-mNG and its mutants at different time points after induction of ER stress by addition of 5 μg/ml Tunicamycin. Cells are treated with 25 nM doxycycline for 24 h to induce expression of IRE1α-mNG and its mutants. Displayed are the values of $n = 3$ independent experiments and the *P* value. The *P* values were determined by a two-sided Student's *t* test. The data are shown as box plots. The central line in the box plot marks the median and the cross shows the mean, the boxes mark the first and third quartiles and the whiskers display the maximum and the minimum points. (**E**) qRT-PCR to monitor CHOP mRNA levels in cells expressing IRE1α-mNG and its mutants (400 nM doxycycline) at different time points after induction of ER stress by addition of 5 μg/ml Tunicamycin. Displayed are the values of $n = 4$ independent experiments.

© The Author(s)
